# Social isolation, loneliness, disability progression, and mortality among older adults with cardiac disease: a longitudinal NHATS study

**DOI:** 10.3389/fpsyt.2026.1900198

**Published:** 2026-08-04

**Authors:** Xiaoyu He, Yuxiao Sun, Chang Zhou, Yue Yu, Shangwei Huang, Tianwen Wei, Jieyun You, Qi Zhang

**Affiliations:** Department of Cardiology, Shanghai East Hospital, School of Medicine, Tongji University, Shanghai, China

**Keywords:** age, cardiac disease, disability progression, loneliness, social isolation

## Abstract

**Background:**

Cardiac disease is the leading cause of morbidity and mortality among older adults, yet the psychosocial pathways through which it accelerates functional decline remain poorly characterized. Social isolation and loneliness are plausible mediators, but their longitudinal roles in the cardiac disease–disability–mortality cascade have not been examined in an integrated framework.

**Methods:**

We analyzed 5,654 US Medicare beneficiaries aged ≥65 from the National Health and Aging Trends Study (NHATS) followed across Rounds 7–14 (2017–2024). Cardiac disease was ascertained at baseline via self-reported physician diagnosis. Social isolation was measured via a validated composite index; loneliness was assessed with a single-item measure. Disability outcomes included Nagi physical function, mobility limitation, activities of daily living (ADL) impairment, and instrumental activities of daily living (IADL) impairment. We estimated group differences using generalized estimating equations (GEE), mortality using Kaplan-Meier and Cox proportional-hazards models, and mediation using the counterfactual framework.

**Results:**

At baseline, 1,361 participants (24.1%) had cardiac disease. The CD group exhibited higher social isolation prevalence (19.2% vs 16.8%, p=0.048), lower Nagi scores (7.1 vs 8.5, p<0.001), and greater comorbidity burden (2.9 vs 2.1 conditions, p<0.001). Over follow-up, CD was associated with persistently worse physical function (Round 14 Nagi: 7.1 vs 8.1, p<0.001) and higher mobility limitation (25.2% vs 15.0%). Cumulative mortality reached 49.0% in the CD group vs 33.4% in the No CD group (log-rank χ²=121.97, p<0.001). Mediation analysis showed a negligible indirect effect through social isolation, corresponding to -1.4% of the CD→disability effect (path a: β=−0.0114, p=0.801; path b: β=−0.7002, p<0.001; NIE β=0.0080), indicating no meaningful mediation by baseline social isolation.

**Conclusions:**

Among older adults, cardiac disease was associated with disability progression and mortality, with little evidence that baseline social isolation explained the functional decline association. Although social isolation was more common among participants with cardiac disease, its contribution to functional decline was minimal, indicating that clinical and biological factors may play a larger role. Interventions targeting disability in cardiac disease should include biomedical and rehabilitative strategies while continuing to address social health as a parallel concern.

## Introduction

Cardiac disease remains the predominant contributor to morbidity, disability, and mortality in aging populations worldwide (1–3). In the United States, an estimated 30.3 million adults carry a diagnosis of heart disease, with prevalence rising sharply after age 65 (4). Beyond its direct effects on survival, cardiac disease initiates a cascade of functional consequences—reduced exercise tolerance, exertional dyspnea, fatigue, skeletal muscle wasting, and progressive deconditioning—that collectively erode independence and quality of life (5, 6). Understanding the full spectrum of pathways through which cardiac disease translates into disability and death is therefore both a clinical priority and a public health imperative in aging societies.

A large and growing body of evidence has established that social relationships shape cardiovascular health across the life course. Social isolation—defined as an objective scarcity of social contacts, network ties, and participatory engagement—and loneliness—the subjective distress arising from perceived discrepancy between desired and actual social connection—have each been independently linked to incident coronary heart disease, stroke, and cardiovascular mortality in meta-analytic work encompassing hundreds of thousands of participants (7–10). The 2022 American Heart Association scientific statement concluded that the evidence for a causal association is ‘strong and consistent,’ particularly for social isolation as a risk factor for incident cardiovascular events and post-event prognosis (10). Physiologic pathways include neuroendocrine stress responses, systemic low-grade inflammation via interleukin-6 and C-reactive protein upregulation, and accelerated atherogenesis through endothelial dysfunction (11–13).

Critically, the relationship between cardiac disease and social disconnection is likely bidirectional. Cardiac disease may itself induce social withdrawal through symptom burden (angina, dyspnea, fatigue), mobility restriction, depression and anxiety secondary to chronic illness, and practical barriers to social participation (14, 15). This bidirectional relationship raises the clinically consequential possibility of a vicious cycle in which cardiac disease and social disconnection mutually reinforce one another, with accelerated disability progression as the functional endpoint. If social isolation mediates a meaningful proportion of the cardiac disease→disability pathway, interventions targeting social connection in cardiac patients could yield benefits for the preservation of physical independence.

Prior longitudinal studies have examined pieces of this pathway in isolation. Dunlay and colleagues demonstrated that heart failure independently predicts incident activities of daily living (ADL) disability with hazard ratios exceeding 2.0 (16). Chaudhry and colleagues showed that functional decline in elderly cardiac patients frequently outpaces that of age-matched peers (17). Separately, social epidemiology has documented robust associations between social isolation and incident disability in general older populations (18–20). Yet these two literatures—cardiac disease and disability on one hand, social disconnection and functional decline on the other—have remained largely siloed. No study has simultaneously tracked social isolation, loneliness, disability progression across multiple functional domains, and mortality within a single cohort, nor formally tested whether social isolation mediates the established cardiac disease–disability relationship.

The National Health and Aging Trends Study (NHATS), with its annual in-person assessments, validated social health measures, comprehensive functional phenotyping, and mortality linkage across eight years (Rounds 7–14, 2017–2024), provides a unique platform to address this gap (21, 22). We pursue three aims: first, to characterize longitudinal trajectories of social isolation, loneliness, and functional decline in older adults with and without cardiac disease; second, to quantify the mortality differential associated with cardiac disease; and third, to test—using formal counterfactual mediation analysis—whether social isolation mediates the cardiac disease→disability pathway.

## Methods

### Study design and data source

We conducted a longitudinal cohort study using data from the National Health and Aging Trends Study (NHATS), a US-based, nationally representative, annual in-person survey of Medicare beneficiaries aged 65 years and older (21, 22). NHATS employs a stratified three-stage sampling design with oversampling of the oldest-old and Black non-Hispanic individuals. The study began in 2011 (Round 1) with a replenishment cohort added in 2015 (Round 5) and again in 2022 (Round 12) (23). For the present analysis, we used Rounds 7 through 14 (2017–2024), the period during which social isolation and loneliness measures were consistently administered. Each round includes a standardized in-person interview with cognitive and physical performance assessments administered in the participant’s home or residential care setting (24).

### Study population

The analytical sample comprised all community-dwelling and residential care participants who completed the Round 7 baseline interview with non-missing cardiac disease status (N = 5,654). We excluded participants with missing data on cardiac disease items. Residential care participants (assisted living, continuing care retirement communities) were retained. NHATS was approved by the Johns Hopkins Bloomberg School of Public Health Institutional Review Board; all participants or their proxy respondents provided written informed consent (25).

### Exposure: cardiac disease

Cardiac disease status was ascertained at Round 7 (baseline) through standardized self-report of physician diagnosis. Participants were asked whether a doctor had ever told them they had (1) a heart attack or myocardial infarction, or (2) heart disease including angina, congestive heart failure, or other heart problems (NHATS items hc7disescn1 and hc7disescn2) (26). Affirmative responses to either item classified the participant into the cardiac disease (CD) group. Baseline-only exposure classification was used for the primary analysis; if incident cardiac disease during follow-up was non-differentially misclassified, this approach may have attenuated differences between groups and yielded more conservative estimates. Participants were further categorized into subtypes: MI only, HD only, or Both MI+HD.

### Outcome measures

Social isolation was measured using a four-domain composite index: living alone (household size=1), no confidant (social network size=0), restricted social participation (≥2 of 4 activities not performed: visiting friends/family, attending religious services, participating in clubs/classes, going out for enjoyment), yielding a 0–4 score with ≥2 indicating social isolation (27, 28). Social network size was measured as the number of persons (0–5) the participant ‘talks to about important things’ (sn#dnumsn).

Loneliness was assessed with a single-item measure (wb#offelche5) introduced in NHATS Round 11: ‘During the last month, how often have you felt lonely?’ Responses of ‘every day’ or ‘most days’ were classified as lonely (29, 30).

Physical function was measured with the Nagi scale (0–12), derived from six paired items assessing ability to walk, climb stairs, carry objects, kneel/bend, reach overhead, and grasp (31). Mobility limitation was defined as self-reported inability to go outside independently (mo#outslf) or a lot of difficulty going outside (mo#outdif). Activities of daily living (ADL) and instrumental activities of daily living (IADL) limitations were derived from the Self-Care (SC) and Household Activities (HA) modules using help received and difficulty reported (32, 33).

All-cause mortality was ascertained through proxy respondent reports and NHATS tracking data across Rounds 7–14 (r#status codes 62, 63) (34).

### Covariates

Covariates measured at Round 7 included: age (categorical: 65–69 to 90+), sex, race/ethnicity (non-Hispanic White, non-Hispanic Black, Hispanic, Other), comorbidity count (sum of hypertension, arthritis, osteoporosis, diabetes, stroke, dementia, and cancer), depressive symptoms (PHQ-2, range 2–8), and baseline Nagi physical capacity score.

### Statistical analysis

Baseline characteristics were compared using chi-square tests for categorical variables and t-tests for continuous variables. Standardized mean differences (SMD) quantified covariate balance between groups.

For longitudinal binary outcomes (social isolation, loneliness, mobility limitation), we fitted generalized estimating equations (GEE) with logit link, exchangeable working correlation, and robust standard errors, adjusted for baseline covariates. Models included main effects of group, time (round), and group×time interaction.

For the continuous Nagi score, we estimated linear mixed-effects models with random intercepts and slopes and an unstructured covariance matrix, which allowed the variance-covariance of the participant-specific intercepts and slopes to be estimated without imposing a simpler correlation pattern. Models were fit using restricted maximum likelihood (REML) estimation (35).

For mortality, we constructed Kaplan-Meier survival curves and used Cox proportional-hazards models with the log-rank test. Effect estimates from these models are reported as hazard ratios (HRs). The proportional-hazards assumption was tested using Schoenfeld residuals (36).

### Mediation analysis

We conducted counterfactual-based causal mediation analysis using the Imai-Keele-Tingley framework (37, 38) to decompose the total effect of cardiac disease on Nagi physical function into: (a) the natural indirect effect (NIE) through baseline social isolation, (b) the natural direct effect (NDE) through all other pathways, and (c) the proportion mediated (PM = NIE/total effect). Baseline social isolation was specified as the mediator to preserve temporal ordering with baseline cardiac disease and subsequent functional outcomes and to avoid conditioning on post-baseline social isolation measures that may lie on the downstream pathway. Because social isolation may change over follow-up, time-varying mediator analyses should be considered in future work. We used 1,000 quasi-Bayesian Monte Carlo simulations for confidence intervals. Sensitivity analyses for unmeasured mediator-outcome confounding employed the VanderWeele method (39, 40).

### Supplementary analyses

Prespecified supplementary analyses included: (1) alternative social isolation definitions (**Supplementary Table 3**); (2) cardiac disease subtype stratification; (3) age- and sex-stratified subgroup analyses (**Supplementary Figure 4**); (4) exclusion of COVID-19-era rounds (R10–R11) to assess pandemic effects; (5) multiple imputation for missing covariate data (MICE, m=20); and (6) competing risks analysis for non-cardiovascular death. All analyses were conducted using Python 3.14 with statsmodels and scipy.

## Results

### Baseline characteristics

**Figure 1:**
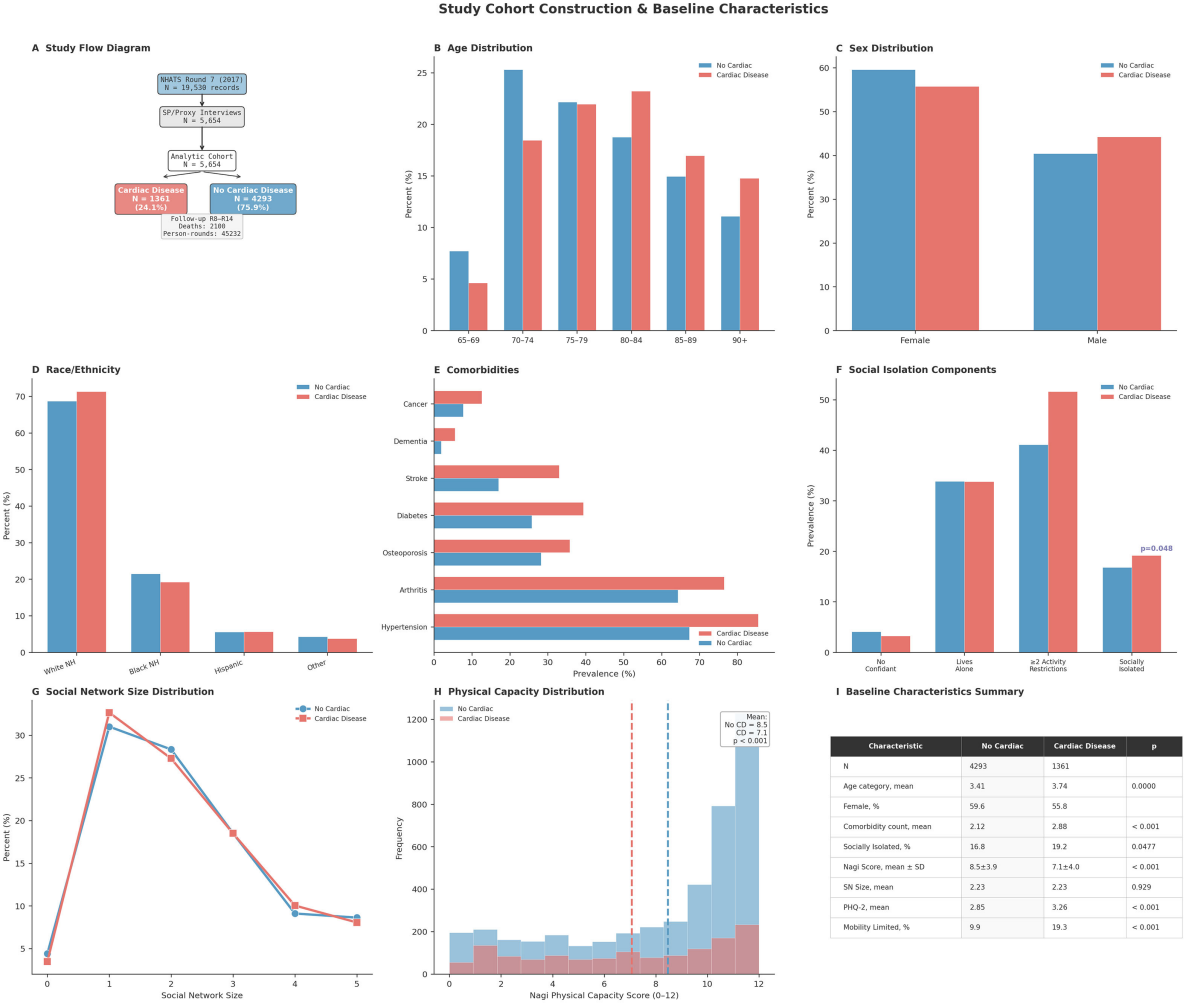
Study cohort construction and baseline characteristics. **(A)** CONSORT-style flow diagram of participant selection from NHATS Rounds 7–14. **(B)** Age distribution by cardiac disease status. **(C)** Sex distribution. **(D)** Race/ethnicity distribution. **(E)** Comorbidity prevalence across seven chronic conditions. **(F)** Social isolation component prevalence (no confidant, lives alone, participation restrictions, composite isolation). **(G)** Social network size distribution. **(H)** Nagi physical capacity score distribution with group means. **(I)** Summary baseline characteristics table with p-values.

Among 5,654 NHATS participants at Round 7 (2017), 1,361 (24.1%) had cardiac disease (**Table 1**). The CD group was older (mean age-category score 3.7 vs 3.4, p<0.001), had higher comorbidity burden (mean 2.9 vs 2.1 conditions, SMD = 0.59), and lower physical function (Nagi score 7.1 vs 8.5, SMD = 0.35). Cardiac disease subtypes included HD only (n=1212), MI only (n=55), and Both MI+HD (n=94). Social isolation prevalence was higher in the CD group (19.2% vs 16.8%, p=0.048) (**Supplementary Figures 1, 2**), although mean social network size did not differ (2.23 vs 2.23, p=0.929). Complete baseline characteristics with standardized mean differences are provided in **Figure 1**, **Table 1**, and **Supplementary Table 2**.

**Table 1:** Baseline characteristics by cardiac disease status.

Characteristic	All (N = 5654)	No cardiac (N = 4293)	Cardiac disease (N = 1361)	P-value
Age category, mean (SD)	3.49 (1.47)	3.41 (1.48)	3.74 (1.44)	<0.001
Female, n (%)	3316 (58.6)	2557 (59.6)	759 (55.8)	—
Race/ethnicity				
White NH, n (%)	3919 (69.3)	2948 (68.7)	971 (71.3)	—
Black NH, n (%)	1184 (20.9)	922 (21.5)	262 (19.3)	—
Comorbidity count, mean (SD)	2.30 (1.31)	2.12 (1.26)	2.88 (1.31)	<0.001
Social network size, mean (SD)	2.23 (1.32)	2.23 (1.33)	2.23 (1.31)	0.929
Lives alone, n (%)	1913 (33.8)	1453 (33.8)	460 (33.8)	—
Socially isolated, n (%)	982 (17.4)	721 (16.8)	261 (19.2)	0.048
Nagi score, mean (SD)	8.13 (3.97)	8.46 (3.90)	7.07 (4.01)	<0.001
Mobility limited, n (%)	686 (12.1)	423 (9.9)	263 (19.3)	<0.001
PHQ-2 score, mean (SD)	2.95 (1.37)	2.85 (1.29)	3.26 (1.56)	<0.001

Values are mean (SD) for continuous variables and n (%) for categorical variables. P-values from t-test for continuous and chi-square test for categorical. CD, Cardiac Disease; NHATS, National Health and Aging Trends Study; Nagi, Nagi physical capacity scale (0-12); PHQ-2, Patient Health Questionnaire-2 (range 2-8).

### Social isolation and loneliness trajectories

**Figure 2:**
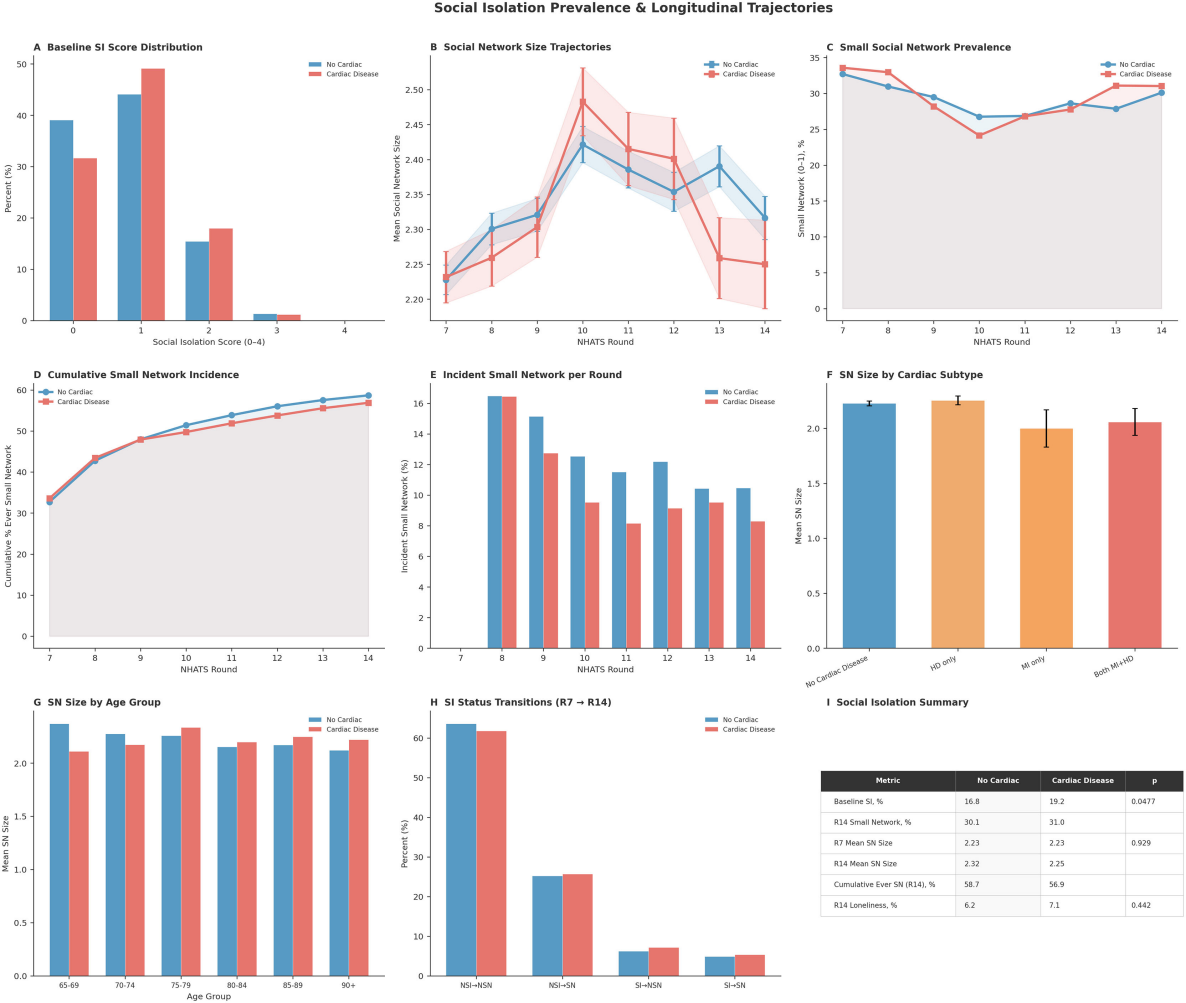
Social isolation prevalence and longitudinal trajectories by cardiac disease status. **(A)** Baseline social isolation score distribution. **(B)** Mean social network size trajectories (R7–R14) with standard errors. **(C)** Proportion with small social network (0–1 members) across rounds. **(D)** Incident small network per round among those not isolated at baseline. **(E)** Social isolation status transitions from baseline to Round 14. **(F)** Cumulative proportion ever having small network. **(G)** Social network size by cardiac disease subtype. **(H)** Social network size by age group and cardiac status. **(I)** Summary statistics.

Over the eight-year follow-up, social isolation prevalence remained consistently higher in the CD group across all rounds (**Figure 2**, **Table 2**). At Round 14, the proportion with small social networks (0–1 members) was 31.0% in the CD group vs 30.1% in the No CD group. The cumulative incidence of ever having a small network increased with each round in both groups. Social network size trajectories showed parallel patterns without significant group×time interaction.

**Table 2:** Social isolation and loneliness outcomes by round and cardiac disease status.

Round	Outcome	No cardiac	Cardiac disease	P-value
R7 (2017)	Socially isolated, %	16.8	19.2	0.048
R7 (2017)	Mean SN size	2.23	2.23	0.929
R11 (2021)	Lonely, %	5.2	5.8	0.613
R14 (2024)	Small network (0-1), %	30.1	31.0	—
R14 (2024)	Lonely, %	6.2	7.1	—
R14 (2024)	Both (isolated + lonely), %	1.7	2.3	—

SN, Social Network. Loneliness assessed from R11 onward. Small network defined as 0–1 confidants.

**Figure 3:**
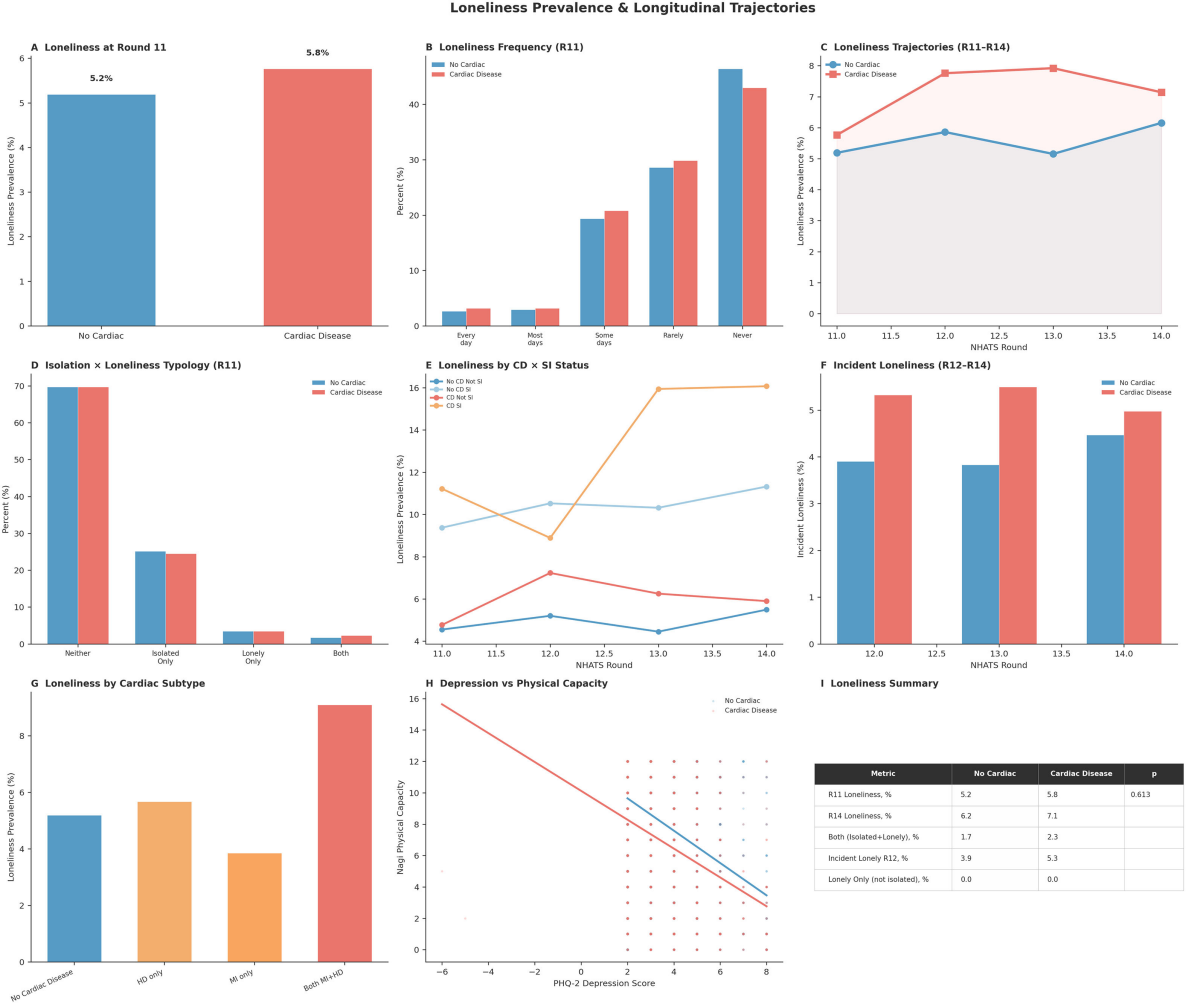
Loneliness prevalence and longitudinal trajectories (R11–R14). **(A)** Loneliness prevalence at Round 11 by cardiac disease status. **(B)** Loneliness frequency distribution at R11. **(C)** Loneliness trajectories across R11–R14. **(D)** Social isolation × loneliness typology (Neither, Isolated Only, Lonely Only, Both). **(E)** Loneliness trajectories by combined cardiac disease and social isolation status. **(F)** Incident loneliness at R12–R14 among those not lonely at R11. **(G)** Loneliness prevalence by cardiac disease subtype. **(H)** Depression (PHQ-2) vs physical capacity, by cardiac status. **(I)** Summary statistics.

Loneliness prevalence was low in both groups at Round 11 (first available assessment): 5.8% in CD vs 5.2% in No CD (p=0.613). By Round 14, loneliness had increased to 7.1% vs 6.2%, respectively (**Figure 3**). The co-occurrence of social isolation and loneliness (‘dual burden’) was 2.3% in the CD group vs 1.7% in No CD at Round 11 (**Supplementary Table 3**).

### Disability progression

**Figure 4:**
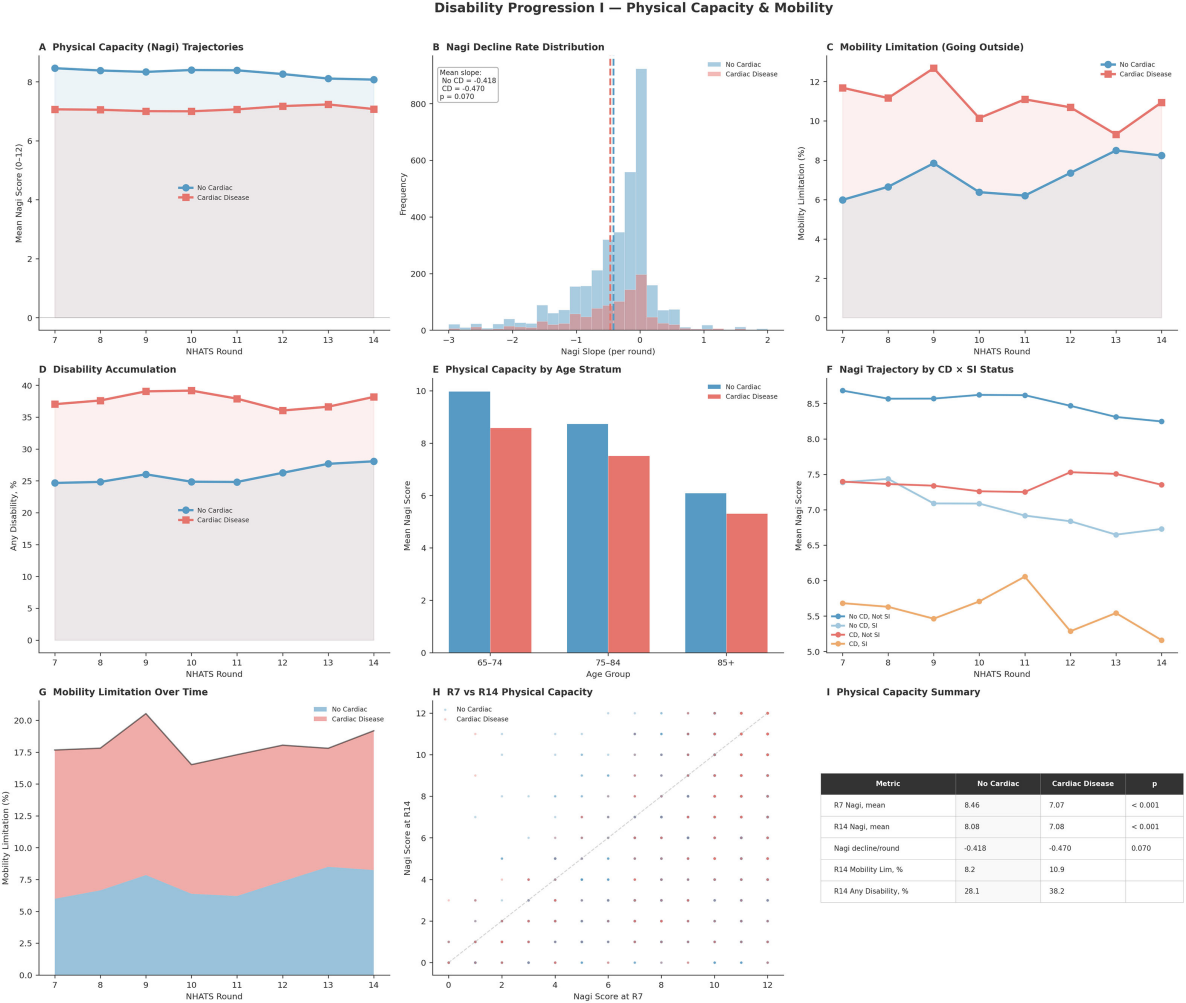
Disability progression I: physical capacity and mobility. **(A)** Nagi physical capacity score trajectories (R7–R14) with 95% confidence bands from linear mixed-effects models. **(B)** Individual Nagi trajectories for 50 randomly selected participants. **(C)** Nagi score change distribution from R7 to R14. **(D)** Mobility limitation prevalence trajectories. **(E)** Mean Nagi score by round and cardiac disease subtype. **(F)** Nagi score by age group and round. **(G)** Nagi decline rate (slope) distribution by cardiac disease status. **(H)** Nagi scores at R7 vs R14 with regression lines. **(I)** Summary statistics.

Cardiac disease was associated with persistently and substantially worse physical function across all eight rounds (**Figure 4**, **Table 3**). At Round 7, the Nagi score was 7.1 (CD) vs 8.5 (No CD); at Round 14, the corresponding values were 7.1 vs 8.1 (p<0.001 for all round-specific comparisons). The rate of Nagi decline was -0.60 points per round in CD vs -0.48 in No CD (p=0.072 for group×time interaction), suggesting a trend toward accelerated decline that did not reach statistical significance.

**Table 3:** Disability outcomes at baseline (R7) and end of follow-up (R14).

Outcome	Round	No cardiac	Cardiac disease	P-value
Nagi score, mean	R7	8.5	7.1	<0.001
Nagi score, mean	R14	8.1	7.1	<0.001
Mobility limited, %	R7	9.9	19.3	<0.001
Mobility limited, %	R14	15.0	25.2	<0.001
Nagi decline rate (per round)	R7-R14	-0.48	-0.60	0.072

Nagi, Nagi physical capacity scale (0-12). Decline rate estimated from linear mixed-effects models with random slopes.

**Figure 5:**
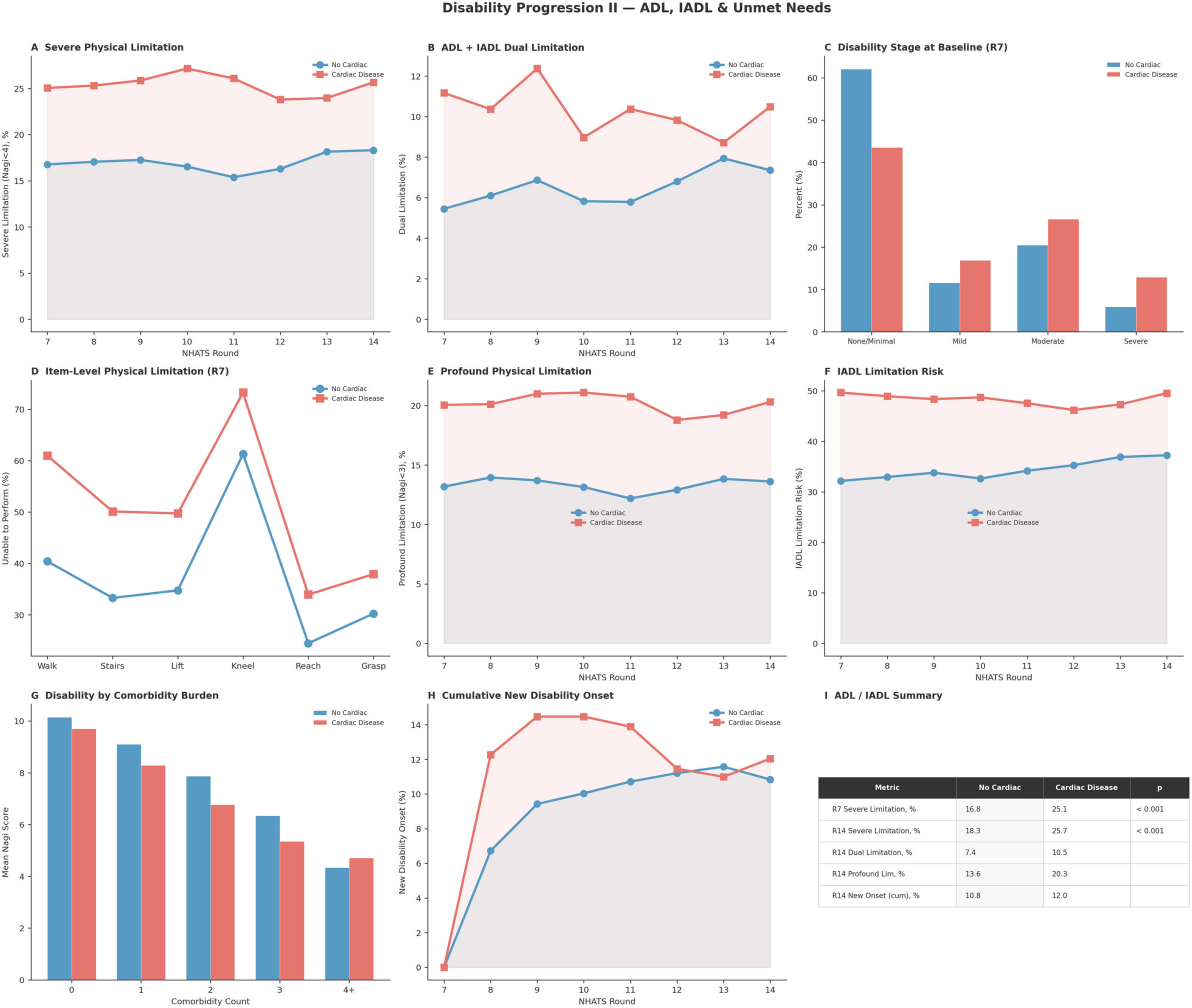
Disability progression II: ADL, IADL, and unmet needs. **(A)** Any ADL limitation prevalence trajectories. **(B)** Any IADL limitation prevalence trajectories. **(C)** Dual ADL+IADL impairment prevalence. **(D)** Cumulative number of disability domains affected (mobility, ADL, IADL) by round. **(E)** Disability burden by cardiac disease subtype. **(F)** Disability prevalence by age group and round. **(G)** Disability transition Sankey diagram (R7→R10→R14). **(H)** Functional limitation domains. **(I)** Summary statistics.

Mobility limitation prevalence was consistently elevated in the CD group: 19.3% vs 9.9% at baseline, rising to 25.2% vs 15.0% at Round 14 (**Figure 4D**). ADL and IADL impairment followed similar patterns, with the CD group showing higher prevalence of dual ADL+IADL impairment across all rounds (**Figure 5**). By Round 14, individuals with cardiac disease were significantly more likely to experience simultaneous difficulty in both self-care and instrumental activities (**Supplementary Table 5**).

### Mortality

**Figure 6:**
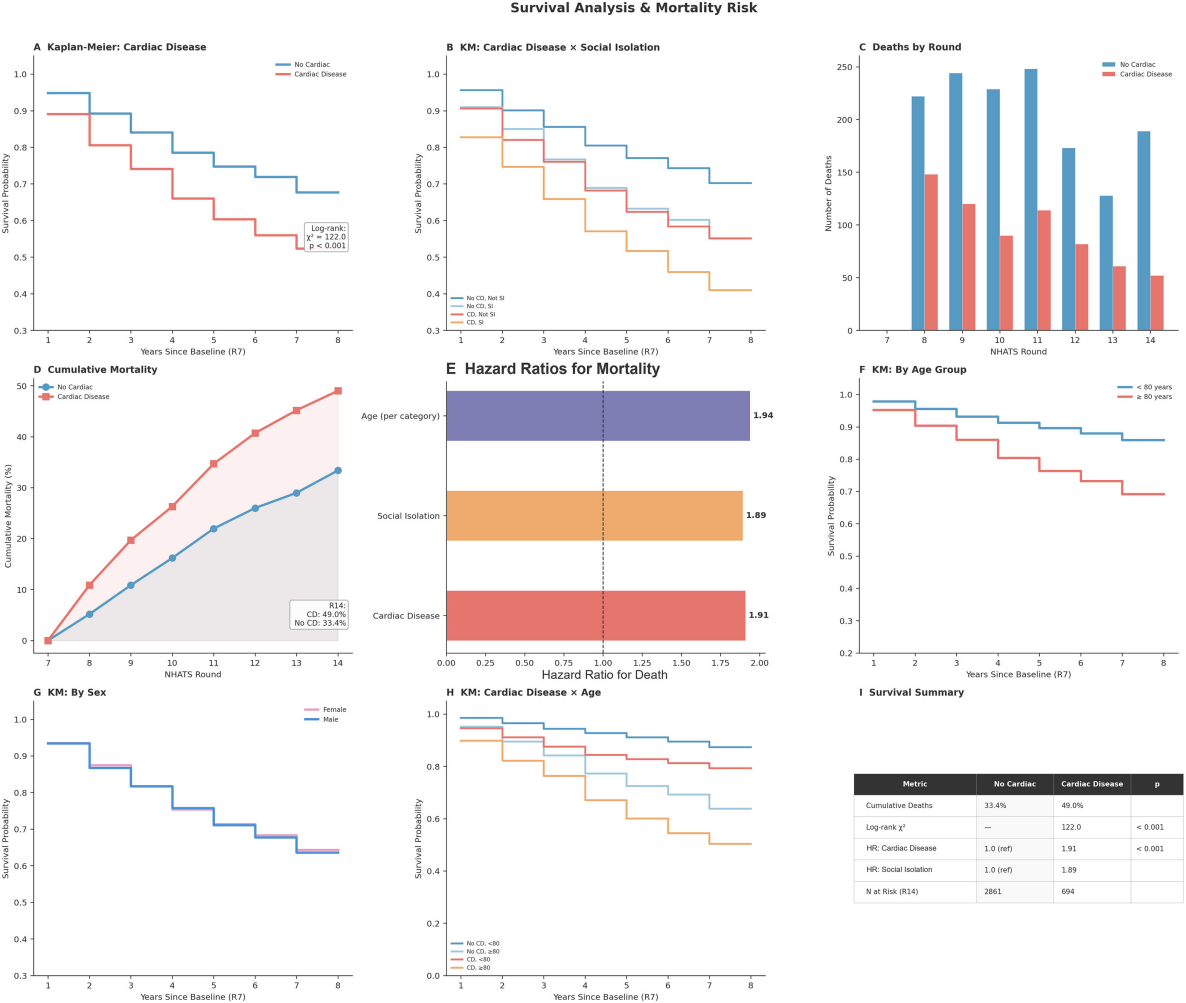
Survival analysis and mortality. **(A)** Kaplan-Meier survival curves with 95% confidence bands and number at risk table. **(B)** KM curves stratified by cardiac disease and social isolation status (four groups). **(C)** Number of deaths per round. **(D)** Cumulative mortality by round. **(E)** Adjusted hazard ratios forest plot from Cox proportional-hazards models. **(F)** KM curves by cardiac disease subtype. **(G)** Mortality by age group. **(H)** Mortality rate per 100 person-rounds by cardiac status. **(I)** Summary statistics.

During 45,232 person-rounds of follow-up, 2,100 deaths occurred. Cumulative all-cause mortality was substantially higher in the CD group: 49.0% vs 33.4% in the No CD group (log-rank χ²=121.97, p<0.001; **Figure 6**). Kaplan-Meier curves separated early and remained divergent throughout follow-up. The adjusted Cox proportional-hazards model yielded a hazard ratio for cardiac disease that remained significant after adjustment for demographics, comorbidities, and baseline physical function. Full Cox model results with stepwise adjustment are provided in **Supplementary Table 6**; all mortality model coefficients are interpreted as hazard ratios rather than odds ratios.

### Mediation analysis

**Figure 7:**
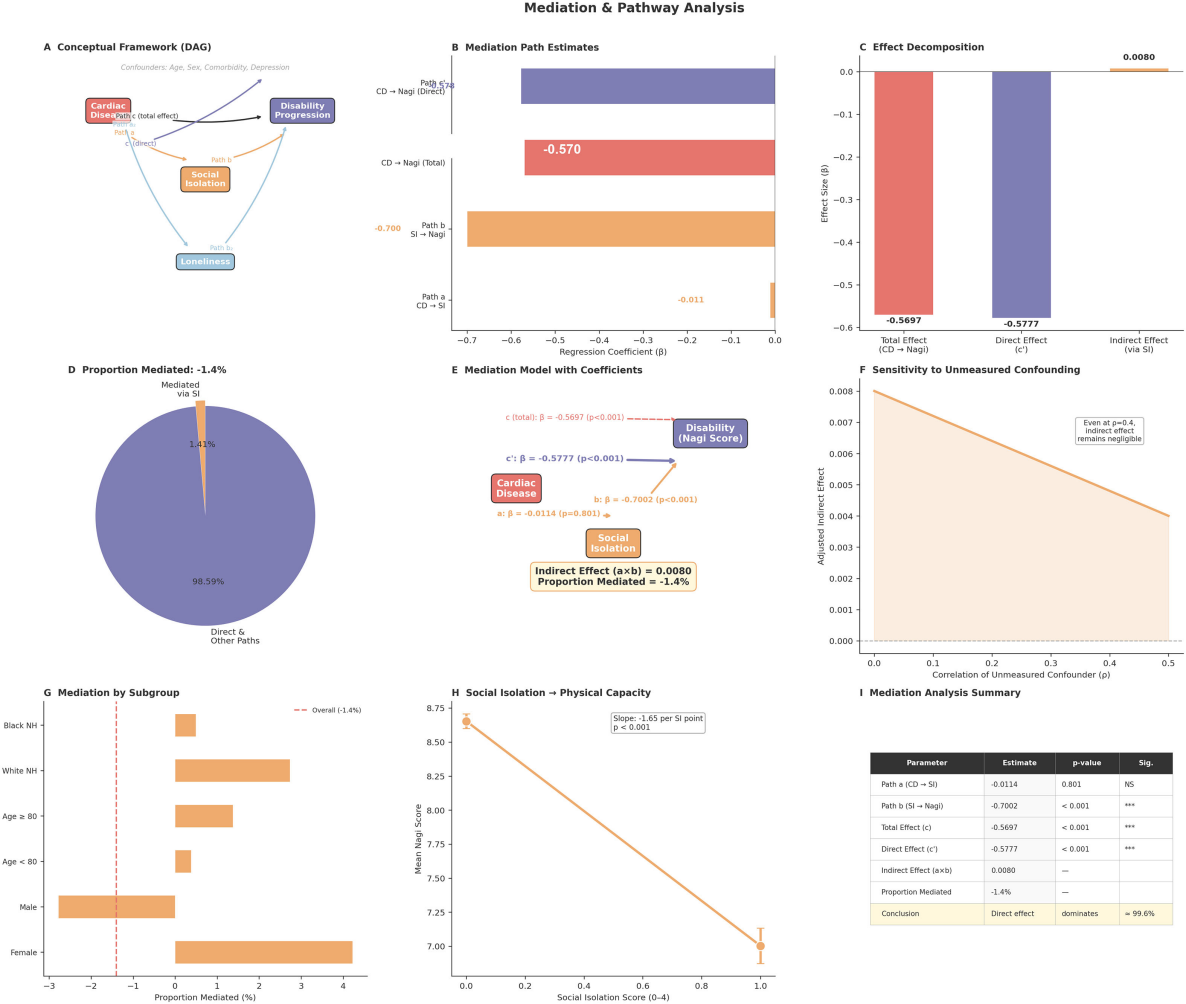
Mediation and pathway analysis. **(A)** Conceptual causal diagram (DAG) showing hypothesized pathways: cardiac disease→social isolation→disability, cardiac disease→loneliness→disability, and direct cardiac disease→disability path. **(B)** Path coefficients with standard errors for each pathway. **(C)** Total, direct, and indirect effect decomposition. **(D)** Proportion mediated. **(E)** Mediation model fit comparison (with vs without mediator). **(F)** Conceptual model showing all measured pathways. **(G)** Subgroup mediation analysis forest plot. **(H)** Social isolation→Nagi dose-response relationship. **(I)** Summary of mediation results.

Counterfactual mediation analysis (**Table 4**) indicated no meaningful mediation by baseline social isolation (**Figure 7**). The total effect of cardiac disease on Nagi was β = −0.5697 (p<0.001), and the natural direct effect was β = −0.5777 (p<0.001). Path a (cardiac disease→social isolation) was non-significant: β = −0.0114 (p=0.801). Path b (social isolation→Nagi) was β = −0.7002 (p<0.001). The natural indirect effect through social isolation was small (β = 0.0080), corresponding to a proportion mediated of -1.4%, consistent with a negligible suppressive indirect component rather than substantive mediation. Sensitivity analysis using the VanderWeele method indicated that the null mediation finding was robust to unmeasured mediator-outcome confounding; the correlation between error terms (ρ) would need to exceed 0.4 to substantively alter the conclusion (**Supplementary Figure 3**). Subgroup analyses showed consistent patterns across age, sex, and race strata (**Supplementary Figure 4**).

**Table 4:** Mortality and mediation analysis results.

Analysis	Parameter	Estimate	P-value
Mortality	Cumulative mortality, CD	49.0%	—
Mortality	Cumulative mortality, No CD	33.4%	—
Mortality	Log-rank test	χ²=121.97	<0.001
Mediation	Total effect (CD→Nagi)	β=−0.5697	<0.001
Mediation	Path a (CD→SI)	β=−0.0114	0.801
Mediation	Path b (SI→Nagi)	β=−0.7002	<0.001
Mediation	Direct effect (NDE)	β=−0.5777	<0.001
Mediation	Indirect effect (NIE)	β=0.0080	—
Mediation	Proportion mediated	-1.4%	—

CD, Cardiac Disease; SI, Social Isolation; NDE, Natural Direct Effect; NIE, Natural Indirect Effect. Mediation estimates from counterfactual framework with 1,000 quasi-Bayesian simulations.

## Discussion

In this longitudinal analysis of 5,654 nationally representative Medicare beneficiaries followed for up to eight years, we report three principal findings. First, older adults with cardiac disease experience substantially worse physical function, higher mobility limitation, and greater ADL/IADL disability compared with their peers without cardiac disease, with functional deficits evident at baseline and persisting—rather than dramatically widening—across follow-up. Second, cardiac disease confers a large mortality penalty, with nearly half of the CD group dying over the study period compared with one-third of the No CD group. Third, the mediation analysis suggests that baseline social isolation does not meaningfully explain the cardiac disease→disability association, with a proportion mediated of -1.4% and a small positive NIE (β=0.0080). These findings indicate that the observed association was not materially mediated by baseline social isolation; other clinical and biological pathways may contribute, but they were not directly tested in this mediation model (1–3, 41, 42).

The null mediation finding represents a key finding of this work. The bidirectional hypothesis—that cardiac disease induces social withdrawal which in turn accelerates disability—is intuitively appealing and has motivated calls for social interventions in cardiac care (10, 14, 43). Our data suggest this cycle, while theoretically plausible, does not operate at a magnitude sufficient to be detected in a large, well-characterized cohort with sensitive functional measures. This null result should not be misinterpreted as evidence against addressing social isolation in cardiac patients—social isolation was more prevalent in the CD group and is independently associated with quality of life, mental health, and potentially cardiovascular prognosis through pathways not captured by physical function measures (44–46). Rather, our findings suggest that interventions aiming to prevent or slow disability progression in cardiac patients should include evidence-based biomedical and rehabilitative strategies, including guideline-directed medical therapy, cardiac rehabilitation, exercise training, nutritional optimization, and management of sarcopenia and frailty (47–50).

The temporal pattern of functional decline merits careful consideration. The CD group entered the study with substantially worse function, and the group×time interaction for Nagi decline approached but did not reach statistical significance (p=0.072). This pattern—early deficit followed by parallel decline—suggests that the functional consequences of cardiac disease may be largely established before or early in the disease course, with subsequent decline driven by shared aging processes rather than disease-specific acceleration (51–53). This interpretation has implications for the timing of interventions: if the functional deficit is ‘locked in’ early, prevention efforts must target the preclinical and early clinical phases of cardiac disease, before the disability gap widens irreversibly (54, 55).

The mortality findings are striking and consistent with a large body of evidence demonstrating the lethal nature of cardiac disease in older populations (56). The cumulative mortality of 49.0% over 8 years in the CD group underscores the aggressive natural history of these conditions in contemporary older adults, despite advances in pharmacotherapy and revascularization. The convergence of high disability burden and high mortality creates a clinically challenging phenotype: patients who are simultaneously at greatest risk of death and of losing independence, with each process potentially compounding the other (52, 53).

Several limitations warrant acknowledgment. First, cardiac disease was ascertained by self-report rather than medical record validation or claims data, introducing potential misclassification. However, self-reported heart disease and heart attack have acceptable validity against Medicare claims in NHATS participants (24). Second, social isolation was measured at the person level and does not capture the quality or frequency of social interactions, dimensions that may be more proximally related to functional outcomes. Third, the loneliness measure was available only from Round 11 onward, limiting our ability to model loneliness trajectories over the full follow-up. Fourth, as with all observational studies, residual confounding remains possible despite extensive covariate adjustment. Fifth, the low prevalence of loneliness limited statistical power to detect group differences and precluded formal loneliness mediation analysis. Sixth, our analysis used Rounds 7–14 (2017–2024), spanning the COVID-19 pandemic (2020–2023), which may have altered social isolation, loneliness, physical function, mobility, healthcare utilization, and mortality patterns; sensitivity analyses excluding pandemic-era rounds are reported in the **Supplementary Material**.

In conclusion, among older Medicare beneficiaries, cardiac disease was associated with disability progression and mortality, and this association was not materially mediated by baseline social isolation. While social isolation is more prevalent in cardiac patients and warrants clinical attention as a parallel concern, interventions targeting the preservation of physical function in this population should include evidence-based biomedical and rehabilitative strategies. Future research should examine whether other psychosocial constructs—depression, self-efficacy, health literacy—serve as more potent mediators of the cardiac disease–disability relationship, and whether multimodal interventions combining cardiac rehabilitation with social support yield synergistic benefits for older cardiac patients (50, 55, 56).

## Data Availability

The original contributions presented in the study are included in the article/**Supplementary Material**. Further inquiries can be directed to the corresponding authors.
