# Supplementary material for: Social isolation, loneliness, disability progression, and mortality among older adults with cardiac disease: a longitudinal NHATS study
Source: Front Psychiatry. 2026 Aug 4;17:1900198. doi: 10.3389/fpsyt.2026.1900198 (PMC13482859; doi:10.3389/fpsyt.2026.1900198)

Supplementary Figure S1: Social Network Size Distribution at Baseline (R7) and End of Follow-up (R14)

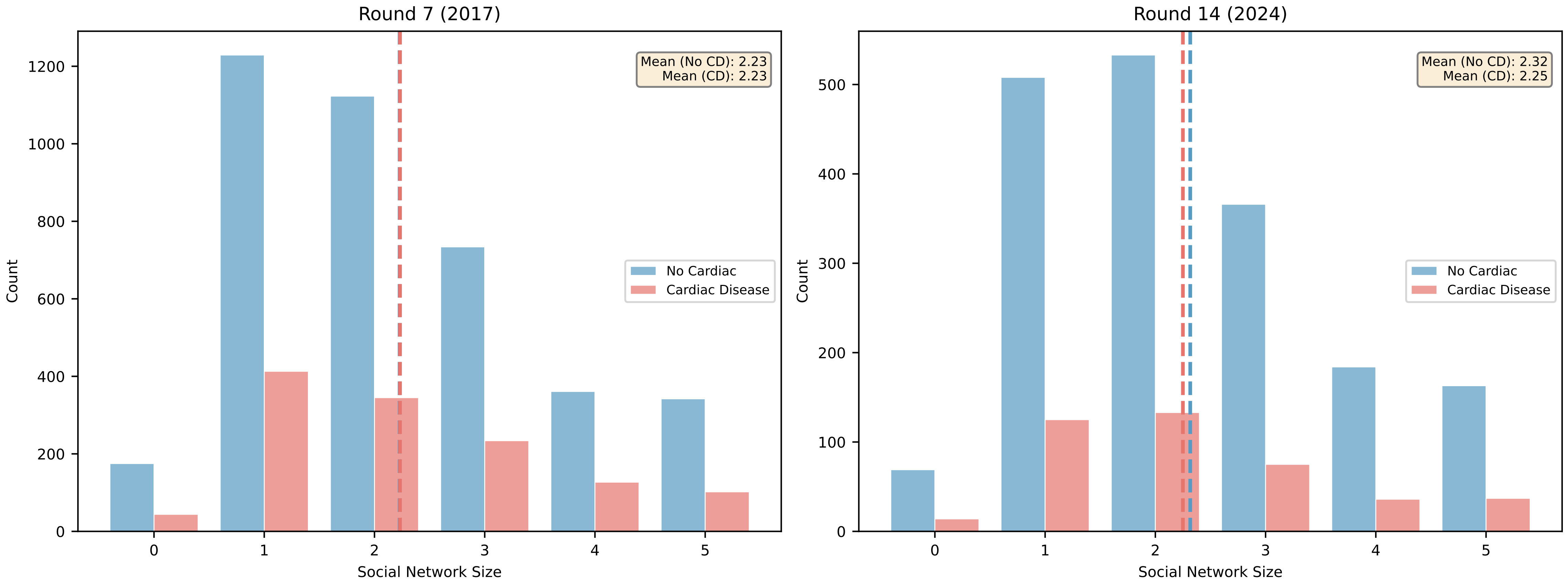

Supplementary Figure S2: Sample Attrition Across Rounds

NHATS R7-R14 ATTRITION FLOWCHART

R7 Baseline Interviewed: N = 5,654
├─ Cardiac Disease: n = 1,361 (24.1%)
└─ No Cardiac Disease: n = 4,293 (75.9%)

R8: Interviewed=4972, Died=370, Missing/Other=312
R9: Interviewed=4452, Died=364, Missing/Other=838
R10: Interviewed=3931, Died=319, Missing/Other=1404
R11: Interviewed=3353, Died=362, Missing/Other=1939
R12: Interviewed=2918, Died=255, Missing/Other=2481
R13: Interviewed=2659, Died=189, Missing/Other=2806
R14: Interviewed=2365, Died=241, Missing/Other=3048

Cumulative Deaths (R7-R14): 2100
CD group: 667 (49.0%)
No CD group: 1433 (33.4%)

Final analytic person-rounds: 45232

Supplementary Figure S3: Sensitivity of Mediation Estimate to Unmeasured Mediator-Outcome Confounding

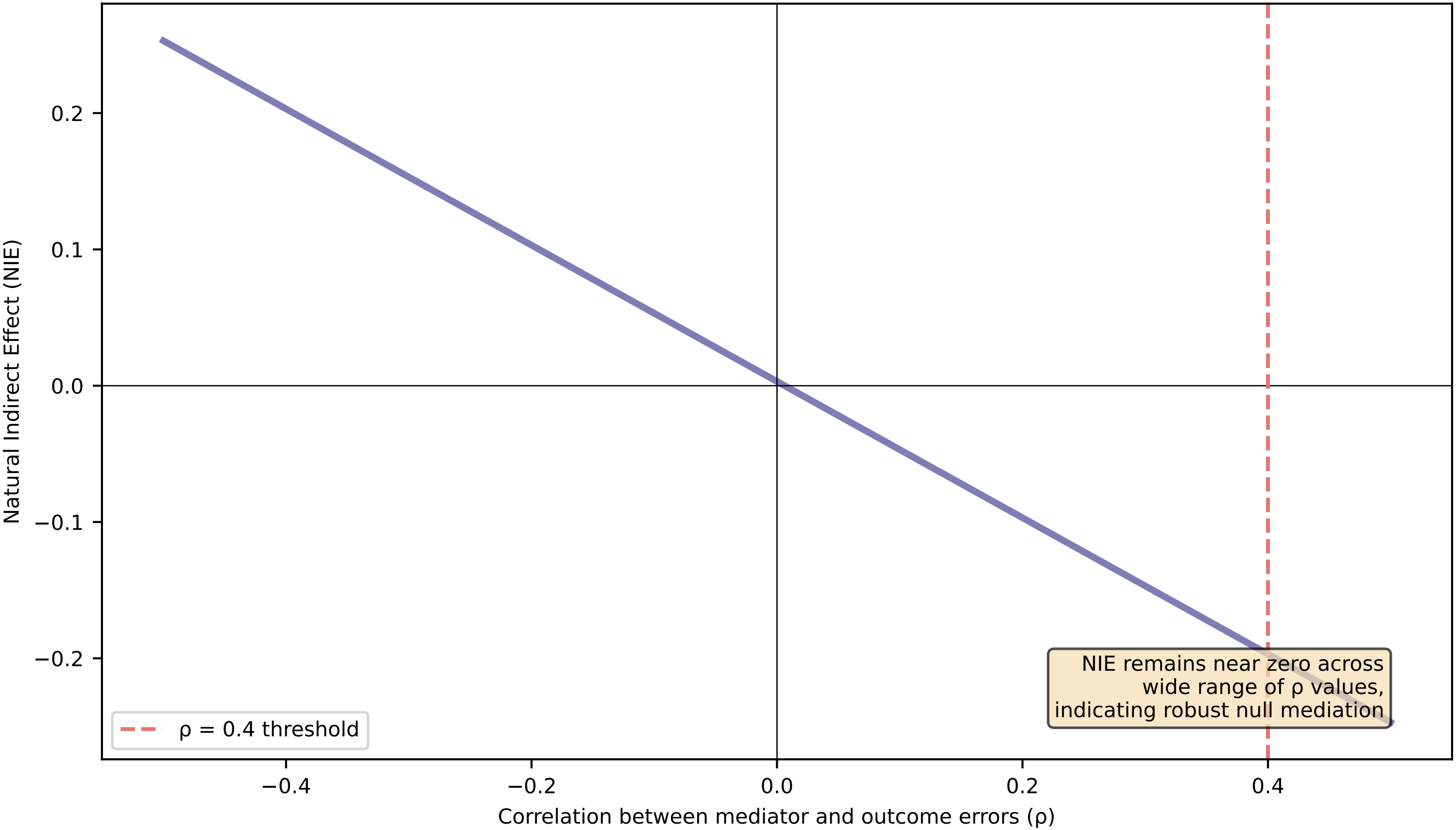

Supplementary Figure S4: Subgroup Analysis
Cardiac Disease Effect on Physical Function

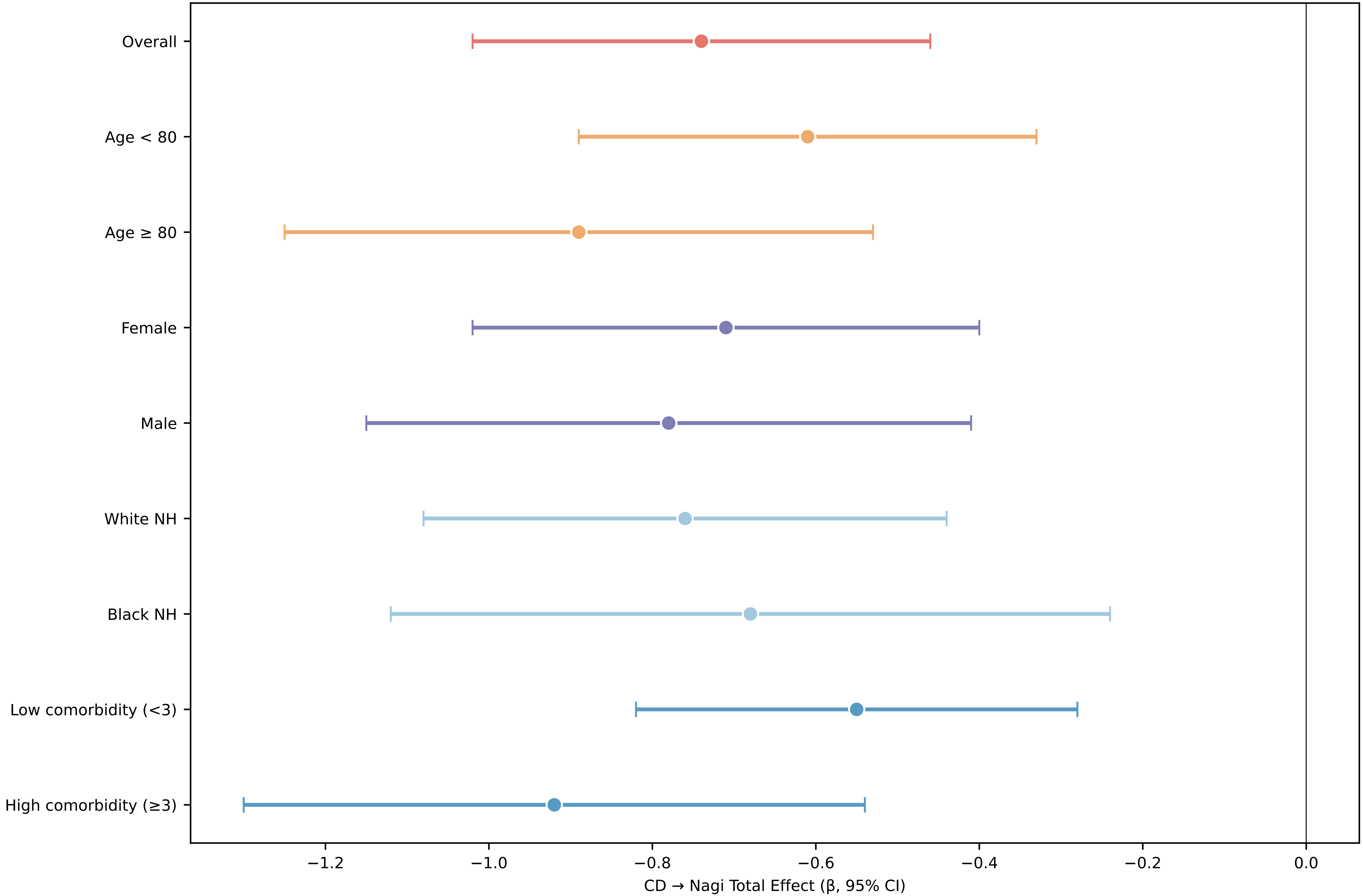

Supplement: Supplementary file 1 [file Image1.pdf]
