# Supplementary material for: Social isolation, loneliness, disability progression, and mortality among older adults with cardiac disease: a longitudinal NHATS study
Source: Front Psychiatry. 2026 Aug 4;17:1900198. doi: 10.3389/fpsyt.2026.1900198 (PMC13482859; doi:10.3389/fpsyt.2026.1900198)
Supplement: Supplementary file 2 [file Table1.docx]

**Supplementary Table S1. Sample Selection from NHATS Rounds 7–14.**

| **Selection Criterion** | **N** |
| --- | --- |
| Total Round 7 records in NHATS data file | 19530 |
| Excluded: Not interviewed at Round 7 | 13876 |
| Died before Round 7 interview | 500 |
| Non-response / unable to contact / other | 636 |
| Not in sample (-1) | 12740 |
| Included: Completed Round 7 interview (SP or proxy) | 5,654 |
| SP interview (status=60) | 5525 |
| Proxy interview (status=61) | 129 |
| Analytic sample (cardiac disease status available) | 5,654 |
| Cardiac disease group | 1,361 (24.1%) |
| No cardiac disease group | 4,293 (75.9%) |

*S1 Footnote: NHATS = National Health and Aging Trends Study. SP = sample person. Proxy interviews conducted when SP unable to respond due to cognitive or health limitations.*

**Supplementary Table S2. Baseline Characteristics of NHATS Participants by Cardiac Disease Status with Standardized Mean Differences.**

| **Characteristic** | **No Cardiac Disease (n=4,293)** | **Cardiac Disease (n=1,361)** | **SMD** | **P-value** |
| --- | --- | --- | --- | --- |
| Total N | 4,293 | 1,361 |  |  |
| Age category, mean (SD) | 3.41 (1.48) | 3.74 (1.44) | 0.223 | <0.001 |
| Female, n (%) | 2557 (59.6%) | 759 (55.8%) | 0.077 | 0.012 |
| 65–69 years | 331 (7.7%) | 63 (4.6%) |  |  |
| 70–74 years | 1086 (25.3%) | 251 (18.4%) |  |  |
| 75–79 years | 952 (22.2%) | 299 (22.0%) |  |  |
| 80–84 years | 806 (18.8%) | 316 (23.2%) |  |  |
| 85–89 years | 642 (15.0%) | 231 (17.0%) |  |  |
| ≥90 years | 0 (0.0%) | 0 (0.0%) |  |  |
| Race/ethnicity |  |  |  |  |
| White, non-Hispanic | 2948 (68.7%) | 971 (71.3%) |  |  |
| Black, non-Hispanic | 922 (21.5%) | 262 (19.3%) |  |  |
| Hispanic | 240 (5.6%) | 77 (5.7%) |  |  |
| Other | 183 (4.3%) | 51 (3.7%) |  |  |
| Comorbidity count, mean (SD) | 2.12 (1.26) | 2.88 (1.31) | 0.590 | <0.001 |
| Hypertension | 2890 (67.3%) | 1163 (85.5%) | 0.437 |  |
| Arthritis | 2761 (64.3%) | 1041 (76.5%) | 0.269 |  |
| Osteoporosis | 1211 (28.2%) | 487 (35.8%) | 0.163 |  |
| Diabetes | 1107 (25.8%) | 536 (39.4%) | 0.293 |  |
| Stroke | 730 (17.0%) | 449 (33.0%) | 0.376 |  |
| Dementia/Alzheimer | 82 (1.9%) | 75 (5.5%) | 0.191 |  |
| Cancer (excluding skin) | 329 (7.7%) | 171 (12.6%) | 0.163 |  |
| Social network size, mean (SD) | 2.23 (1.33) | 2.23 (1.31) | 0.003 | 0.929 |
| No confidant, n (%) | 175 (4.1%) | 44 (3.2%) | 0.045 |  |
| Lives alone, n (%) | 1453 (33.8%) | 460 (33.8%) | 0.001 |  |
| Socially isolated, n (%) | 721 (16.8%) | 261 (19.2%) | 0.062 | 0.048 |
| Nagi score, mean (SD) | 8.46 (3.90) | 7.07 (4.01) | 0.353 | <0.001 |
| Mobility limitation, n (%) | 423 (9.9%) | 263 (19.3%) | 0.271 | <0.001 |
| PHQ-2 score, mean (SD) | 2.85 (1.29) | 3.26 (1.56) | 0.287 | <0.001 |
| GAD-2 score, mean (SD) | 2.75 (1.26) | 3.17 (1.59) | 0.290 | <0.001 |

*S2 Footnote: CD = cardiac disease; SMD = standardized mean difference (absolute value). SMD > 0.10 indicates meaningful baseline imbalance. P-values from t-test (continuous) or chi-square test (categorical). PHQ-2 = Patient Health Questionnaire-2; GAD-2 = Generalized Anxiety Disorder-2. Nagi score range 0–12, higher = better physical function.*

**Supplementary Table S3. Social Network Characteristics by Round and Cardiac Disease Status.**

| **Round** | **No CD: Mean SN size (SD)** | **CD: Mean SN size (SD)** | **No CD: Small network (%)** | **CD: Small network (%)** | **CD N** | **No CD N** |
| --- | --- | --- | --- | --- | --- | --- |
| Round 7 | 2.23 (1.33) | 2.23 (1.31) | 35.4% | 36.1% | 1361 | 4293 |
| Round 8 | 2.30 (1.35) | 2.26 (1.33) | 33.4% | 35.0% | 1138 | 3834 |
| Round 9 | 2.32 (1.34) | 2.30 (1.30) | 31.6% | 30.5% | 986 | 3466 |
| Round 10 | 2.42 (1.36) | 2.48 (1.34) | 29.6% | 27.4% | 858 | 3073 |
| Round 11 | 2.39 (1.31) | 2.42 (1.32) | 28.9% | 29.5% | 694 | 2659 |
| Round 12 | 2.35 (1.31) | 2.40 (1.36) | 30.2% | 29.8% | 580 | 2338 |
| Round 13 | 2.39 (1.32) | 2.26 (1.27) | 29.5% | 32.5% | 505 | 2154 |
| Round 14 | 2.32 (1.32) | 2.25 (1.30) | 31.7% | 33.1% | 448 | 1917 |

*S3 Footnote: SN = social network. Small network defined as 0–1 confidants. CD = cardiac disease. Data from NHATS Rounds 7–14 (2017–2024). Only participants with completed interviews included.*

**Supplementary Table S4. Nagi Physical Capacity Item-Level Difficulty Prevalence by Round and Cardiac Disease Status.**

| **Round** | **Nagi Item** | **No CD: Unable (%)** | **CD: Unable (%)** |
| --- | --- | --- | --- |
| R7 | Walk 6 blocks (~1/2 mile) | 0.0% | 0.0% |
| R7 | Walk up 20 stairs | 0.0% | 0.0% |
| R7 | Lift and carry 20 pounds | 0.0% | 0.0% |
| R7 | Kneel down without support | 0.0% | 0.0% |
| R7 | Put heavy object on shelf overhead | 0.0% | 0.0% |
| R7 | Open sealed jar using hands only | 0.0% | 0.0% |
| R14 | Walk 6 blocks (~1/2 mile) | 0.0% | 0.0% |
| R14 | Walk up 20 stairs | 0.0% | 0.0% |
| R14 | Lift and carry 20 pounds | 0.0% | 0.0% |
| R14 | Kneel down without support | 0.0% | 0.0% |
| R14 | Put heavy object on shelf overhead | 0.0% | 0.0% |
| R14 | Open sealed jar using hands only | 0.0% | 0.0% |

*S4 Footnote: CD = cardiac disease. "Unable" defined as response of "no" (cannot do) to each item. Nagi items are paired; harder items shown. R7=baseline (2017), R14=end of follow-up (2024).*

**Supplementary Table S5. Mobility Limitation Prevalence by Round and Cardiac Disease Status.**

| **Round** | **No CD: Mobility limited (%)** | **CD: Mobility limited (%)** | **No CD N** | **CD N** |
| --- | --- | --- | --- | --- |
| Round 7 | 6.0% | 11.7% | 4293 | 1361 |
| Round 8 | 6.7% | 11.2% | 3834 | 1138 |
| Round 9 | 7.8% | 12.7% | 3466 | 986 |
| Round 10 | 6.4% | 10.1% | 3073 | 858 |
| Round 11 | 6.2% | 11.1% | 2659 | 694 |
| Round 12 | 7.4% | 10.7% | 2338 | 580 |
| Round 13 | 8.5% | 9.3% | 2154 | 505 |
| Round 14 | 8.2% | 10.9% | 1917 | 448 |

*S5 Footnote: CD = cardiac disease. Mobility limitation defined as never going outside independently or going outside only some/none of the time. Data from NHATS Rounds 7–14.*

**Supplementary Table S6. Cox Proportional Hazards Models: Cardiac Disease and All-Cause Mortality.**

| **Model** | **HR for CD** | **95% CI** | **P-value** |
| --- | --- | --- | --- |
| Model 1: Unadjusted | 1.72 | (1.58–1.87) | <0.001 |
| Model 2: + Demographics | 1.65 | (1.51–1.80) | <0.001 |
| Model 3: + Comorbidities | 1.52 | (1.38–1.67) | <0.001 |
| Model 4: + Baseline function | 1.47 | (1.33–1.62) | <0.001 |

*S6 Footnote: HR estimated from Cox proportional hazards models. CD = cardiac disease. Full adjustment covariates: age, sex, race/ethnicity, comorbidity count, PHQ-2, baseline Nagi score.*

**Supplementary Table S7. Sensitivity Analysis: Alternative Cardiac Disease Definitions.**

| **Definition** | **CD Prevalence** | **HR for Mortality (95% CI)** | **Nagi β (95% CI)** |
| --- | --- | --- | --- |
| Primary: Baseline-only CD | 24.1% | 1.47 (1.33–1.62) | −0.74 (−0.99 to −0.49) |
| Sensitivity 1: Ever CD (including incident) | 28.3% | 1.51 (1.38–1.65) | −0.81 (−1.07 to −0.55) |
| Sensitivity 2: MI only (excluding HD without MI) | 2.6% | 1.38 (1.12–1.70) | −0.52 (−0.91 to −0.13) |
| Sensitivity 3: HD only (excluding MI) | 21.5% | 1.43 (1.29–1.58) | −0.69 (−0.95 to −0.43) |
| Sensitivity 4: Hypertension + CD combined | 71.7% | 1.21 (1.08–1.35) | −0.45 (−0.68 to −0.22) |

*S7 Footnote: MI = myocardial infarction; HD = heart disease. All models adjusted for baseline covariates. Time-varying exposure reclassifies participants at the round of incident diagnosis.*

**Supplementary Table S8. Sensitivity Analysis: Excluding Participants with Baseline Mobility Limitation.**

| **Analysis** | **N** | **HR for Mortality (95% CI)** | **Nagi β (95% CI)** | **SI Mediation %** |
| --- | --- | --- | --- | --- |
| Primary analysis (all participants) | 5,654 | 1.47 (1.33–1.62) | −0.74 (−0.99 to −0.49) | 0.4% |
| Excluding baseline mobility limitation | 4,968 | 1.52 (1.35–1.71) | −0.61 (−0.88 to −0.34) | 0.6% |
| Excluding baseline ADL disability | 4,821 | 1.49 (1.31–1.69) | −0.58 (−0.86 to −0.30) | 0.5% |

*S8 Footnote: ADL = activities of daily living. Mobility limitation defined as inability to go outside independently.*

**Supplementary Table S9. Subgroup Analysis: Cardiac Disease Effect on Physical Function and Mortality.**

| **Subgroup** | **N** | **Nagi β (95% CI)** | **P-interaction** | **HR for Mortality (95% CI)** | **P-interaction** |
| --- | --- | --- | --- | --- | --- |
| Overall | 5,654 | −0.74 (−0.99 to −0.49) | — | 1.47 (1.33–1.62) | — |
| Age < 80 years | 2,982 | −0.61 (−0.89 to −0.33) | 0.18 | 1.55 (1.32–1.82) | 0.33 |
| Age ≥ 80 years | 2,672 | −0.89 (−1.25 to −0.53) |  | 1.41 (1.24–1.60) |  |
| Female | 3,316 | −0.71 (−1.02 to −0.40) | 0.67 | 1.43 (1.26–1.63) | 0.52 |
| Male | 2,338 | −0.78 (−1.15 to −0.41) |  | 1.52 (1.30–1.78) |  |
| White NH | 3,919 | −0.76 (−1.08 to −0.44) | 0.71 | 1.45 (1.28–1.63) | 0.45 |
| Black NH | 1,184 | −0.68 (−1.12 to −0.24) |  | 1.55 (1.27–1.89) |  |
| Low comorbidity (<3) | 3,240 | −0.55 (−0.82 to −0.28) | 0.04 | 1.62 (1.38–1.90) | 0.09 |
| High comorbidity (≥3) | 2,414 | −0.92 (−1.30 to −0.54) |  | 1.35 (1.18–1.54) |  |

*S9 Footnote: NH = non-Hispanic. P-interaction from group × cardiac disease interaction term. Low comorbidity defined as <3 of 7 chronic conditions; high comorbidity ≥3.*

**Supplementary Table S10. Exploratory Mediation Analysis: Loneliness as Mediator of Cardiac Disease–Disability Pathway (NHATS R11–R14).**

| **Pathway** | **Coefficient (β)** | **95% CI** | **P-value** |
| --- | --- | --- | --- |
| Path a: CD → Loneliness (R11) | 0.012 | −0.018 to 0.042 | 0.432 |
| Path b: Loneliness → Nagi score (R11–R14) | −1.243 | −1.586 to −0.900 | <0.001 |
| Total effect c: CD → Nagi score | −0.812 | −1.084 to −0.540 | <0.001 |
| Direct effect c': CD → Nagi (adjusted) | −0.826 | −1.098 to −0.554 | <0.001 |
| Indirect effect a×b | −0.015 | −0.053 to 0.022 | 0.432 |
| Proportion mediated | 1.8% | −2.7% to 6.3% | 0.441 |

*S10 Footnote: CD = cardiac disease. Loneliness defined as "most days" or "every day" in past month (R11+). Mediation analysis restricted to R11–R14 due to availability of loneliness measure. Counterfactual mediation with 1,000 quasi-Bayesian simulations, adjusted for age, sex, race/ethnicity, comorbidity count, and PHQ-2. Loneliness mediation not statistically significant.*

**Supplementary Table S11. Comparison of Key Findings with Published NHATS-Based Studies on Social Isolation and Disability.**

| **Study** | **NHATS Rounds** | **Population** | **Key Finding** | **Present Study Comparison** |
| --- | --- | --- | --- | --- |
| Cudjoe et al. (2020) J Gen Intern Med | R1–R7 | General ≥65 | SI associated with 1.28× odds of ADL disability | Consistent: CD group has 19.2% vs 16.8% SI |
| Freedman et al. (2021) J Gerontol B | R1–R8 | General ≥65 | SN size decline precedes disability | Partially consistent: SN decline slope similar between groups |
| Pohl et al. (2022) JAMA Netw Open | R1–R9 | General ≥65 | Loneliness 5–7% prevalence | Consistent: 5.2–5.8% loneliness at R11 |
| Gong et al. (2023) BMC Geriatr | R5–R10 | CVD patients | CVD associated with accelerated functional decline | Consistent: CD group Nagi 7.1 vs 8.5 at baseline |
| Present study | R7–R14 | CD vs No CD | Direct effect dominates (99.6%); SI mediates 0.4% | Novel: null mediation finding |

*S11 Footnote: SI = social isolation; CD = cardiac disease; CVD = cardiovascular disease; ADL = activities of daily living; SN = social network. Present study extends prior work by formally testing mediation and integrating mortality outcomes.*
